# Older Adults With Cognitive and/or Physical Impairments Can Benefit From Immersive Virtual Reality Experiences: A Feasibility Study

**DOI:** 10.3389/fmed.2019.00329

**Published:** 2020-01-15

**Authors:** Lora Appel, Eva Appel, Orly Bogler, Micaela Wiseman, Leedan Cohen, Natalie Ein, Howard B. Abrams, Jennifer L. Campos

**Affiliations:** ^1^Faculty of Health, School of Health Policy and Management, York University, Toronto, ON, Canada; ^2^OpenLab, University Health Network, Toronto, ON, Canada; ^3^Faculty of Medicine, University of Toronto, Toronto, ON, Canada; ^4^Department of Psychology, Ryerson University, Toronto, ON, Canada; ^5^KITE, Toronto Rehabilitation Institute, University Health Network, Toronto, ON, Canada; ^6^Department of Psychology, University of Toronto, Toronto, ON, Canada

**Keywords:** non-pharmacological therapy, dementia, head-mounted-display, interventional study, nature, simulation, long-term care, social isolation

## Abstract

**Background:** Older adults living in long term care, rehabilitation hospitals, and seniors' residences often experience reduced mobility, sometimes resulting in confinement indoors and isolation, which can introduce or aggravate symptoms of depression, anxiety, loneliness, and apathy. As Virtual Reality (VR) technologies become increasingly accessible and affordable, there is a unique opportunity to enable older adults to escape their restricted physical realities and be transported to both stimulating and calming places which may improve their general well-being. To date no robust evaluations of the use of immersive VR therapy [experienced through a head-mounted-display (HMD)] for older adults within these settings have been reported. VR-therapy may prove to be a safe, inexpensive, non-pharmacological means of managing depressive symptoms and providing engagement and enjoyment to this rapidly growing demographic.

**Objectives:** Establish whether it is feasible to use immersive VR technology as therapy for older adults who have reduced sensory, mobility and/or impaired cognition. This includes evaluation of tolerability, comfort, and ease of use of the HMD, and of the potential for immersive VR to provide enjoyment/relaxation and reduce anxiety and depressive symptoms.

**Methods:** Sixty-six older adults (mean age 80.5, SD = 10.5) with varying cognitive abilities (normal = 28, mild impairment = 17, moderate impairment = 12, severe impairment = 3, unknown cognitive score = 6), and/or physical impairments, entered a multi-site non-randomized interventional study in Toronto, Canada. Participants experienced 3 to 20 min of 360°-video footage of nature scenes displayed on Samsung GearVR HMD. Data was collected through pre/post-intervention surveys, standardized observations during intervention, and post-intervention semi-structured interviews addressing the VR experience.

**Results:** All participants completed the study with no negative side-effects reported (e.g., No dizziness, disorientation, interference with hearing aids); the average time spent in VR was 8 min and 76% of participants viewed the entire experience at least once. Participants tolerated the HMD very well; most had positive feedback, feeling more relaxed and adventurous; 76% wanted to try VR again. Better image quality and increased narrative video content were suggested to improve the experience.

**Conclusion:** It is feasible and safe to expose older adults with various levels of cognitive and physical impairments to immersive VR within these settings. Further research should evaluate the potential benefits of VR in different settings (e.g., home/community based) and explore better customization/optimization of the VR content and equipment for the targeted populations.

## Introduction

Nearly 400,000 Canadians aged 65 years and older and 30% of Canadians 85 years and older live in long-term care (LTC) residences or assisted living housing (1–3). They often experience comorbid conditions (e.g., sensory, cognitive, motor) that can cause limitations to functional mobility (4–7), reduced freedom (8), limited independence (3), restrictions in broad experiences (9), and social isolation. While loss of independence and isolation are associated with poorer health outcomes across all age groups (10), it may be particularly consequential for frail older adults (11, 12), especially those who are experiencing stressful life course transitions, health problems, and/or disabilities (13). Social isolation and loneliness can accelerate the progression of chronic conditions, lead to depression, anxiety, aggressive behavior, and increase the risk of dementia (by up to 40 percent) (14). Optimizing strategies to increase exposure to new experiences and to reduce feelings of isolation may reduce the ensemble of associated negative effects.

There is growing recognition that exposure to natural environments in particular can have a positive impact on health and well-being (15–18). From a psychological/emotional perspective, views of nature elevate levels of positive feelings (pleasantness, calmness), and reduce negatively toned emotions (fear, anger, and sadness) (19). Regarding physiological manifestations of stress recovery, laboratory and clinical investigations have found that viewing nature settings can produce significant restoration indicated by positive changes in blood pressure, heart activity, muscle tension, and brain electrical activity (16, 20). However, it can be challenging to increase exposure to nature in older adults in LTC settings (21). At the individual level, reduced mobility, lack of energy, fear of falling, pain, and medication use can affect one's ability and desire to participate in physical and/or outdoor activities. Infrastructure that lacks accessibility combined with an already high demand on staff time and weather/seasonal conditions are other barriers that limit excursions. These issues are further exacerbated for those living in colder climates with long harsh winters such as Canada. Reassuringly, however, evidence has shown that if unable to engage directly in a natural environment, even visible exposure to nature scenes can be beneficial. For example, it has been reported that in clinical settings, viewing natural scenery (e.g., ornamental indoor plants or a bedside window view of trees) can reduce hospital length-of-stay and reduce the use of pharmaceuticals (18, 19, 22, 23). It has also been shown that housebound older adults often use a view from their window to stay connected to the outside space that they cannot physically explore (24). Therefore, it would be highly advantageous to identify novel methods of providing older adults with new experiences and exposure to natural scenes, while accounting for barriers to mobility and autonomy as well as concerns for safety.

VR technologies have demonstrated increasing promise as a tool to reduce isolation and increase engagement across a number of populations (25). VR systems consist of technologies that provide the user with sensory information through, for example, visual, auditory, and tactile displays, and can have varying degrees of immersiveness. Visual information is often presented via large projection-based displays, or head-mounted-displays (HMD). Modern HMDs include integrated head tracking, thereby allowing the user to move their head and have their visual perspective change in the virtual environment accordingly. Headphones or loudspeakers can generate spatialized binaural sound. Other interfaces such as joysticks or sensory gloves can provide tactile feedback. The content of immersive VR systems (i.e., virtual environments) can be produced through rendered graphics or through 360 video footage. VR goes beyond traditional display technologies by fully immersing users in an interactive space that they can explore, while eliminating interference from the real world. The types of VR content vary widely, and careful consideration must be given to develop and customize virtual environments that are optimized for the intended user. For instance, in older adults with common cognitive, sensory and/or mobility comorbidities, content must strike a balance that avoids being too under-stimulating or too overstimulating and should align with their personal priorities and interests. As described above, there may also be a unique benefit to virtual environments that mimic scenes of nature, particularly for older adults who have a limited ability to access these natural spaces frequently and independently.

VR exposure therapies have been shown to be effective in the treatment of a variety of conditions including: specific phobias (acrophobia, aviophobia, arachnophobia), social anxiety disorder, public speaking anxiety, panic disorder, posttraumatic stress disorder, acute stress disorder, substance abuse disorders (alcohol and nicotine), and depression (26–30). A number of studies have been published on the use of immersive VR by healthy older adults (31, 32), but very few have empirically examined the feasibility of introducing immersive VR in rehab or long-term care settings (33, 34). Even in studies that do evaluate VR as an intervention for older adults with cognitive and physical impairments (35), these studies most frequently describe VR interventions that involve viewing 3D environments on a computer screen and 3D wall-based projected images, but not HMDs. More recently, anecdotal evidence suggests that VR HMDs may also be an effective way to alleviate stress, depression, and anxiety in seniors with age-related conditions (36–39); however, to date there have been no empirical studies that have formalized and quantified the feasibility and effectiveness of immersive VR (viewed through a HMD) in an older adult population with cognitive and/ or physical impairments.

To summarize, as VR technologies have become increasingly accessible, affordable, and comfortable, there is a unique opportunity to use these technologies to enable older adults with age-related impairments to escape from their often confined realities and be transported to interesting, stimulating, calming, and enjoyable places; experiences that may reduce feelings of boredom, apathy, depression, and isolation and improve overall well-being. It may also promote social interactions by facilitating conversations through storytelling, sharing, or reminiscing with caregivers, family, and friends. Therefore, the overall objective of the current study was to evaluate the feasibility of using immersive VR with a sample of older adults within hospital in- and out-patient settings and long-term care residences, and to determine whether these experiences can be beneficial. These individuals were largely considered to be frail older adults with common cognitive, motor, and sensory comorbidities varying in degree of severity.

We considered participants' tolerance for the VR hardware (i.e., HMD), preference for different VR content/environments, and overall acceptability of the system. We also documented whether any negative side effects were observed (e.g., nausea, dizziness, anxiety, or interference with medical devices) and measured whether the VR experiences were rated as being enjoyable and resulted in changes to anxiety. Outcome measures included pre/post intervention ratings on standardized scales (e.g., State/Trait anxiety scales), as well as transcribed qualitative responses that were provided during and following the VR exposure. Overall, this study provides a critical first step toward exploring the potential of using immersive VR applications to promote well-being in older adults with cognitive, mobility and sensory impairments.

## Methods

### Participants

#### Recruitment Settings and Strategy

Participants were recruited from four locations in Toronto, Canada and represent a diversity of institutions that provide care for older adults: (1) The Day Treatment Center (DTC) at Baycrest Health Sciences, providing a rehabilitation program for older adults living in the community with complex medical conditions, (2) Runnymede Healthcare Centre is a rehabilitation and complex continuing care hospital, (3) Kensington Gardens Health Centre is a long-term care facility providing care to people with Alzheimer's disease and other dementias, and (4) Dotsa Bitove Well-ness Academy is a center providing daily programs for adults with memory loss due to mild-to-moderate dementia, their families and their care partners. While these sites cater to individuals with varying social, cognitive, emotional, and physical needs, most of their clients are older adults who are experiencing declines in their ability to live independently.

The screening approach at all sites employed a purposive sampling design. Each study site had an appointed site research coordinator (RC), a healthcare professional (Registered Nurse, Activationists, Therapeutic Recreation Specialists) who would initially identify eligible participants and indicate to the research assistants (RAs) which individuals were interested. The RA would then explain the details of the study to potential participants and obtain informed consent. A shared decision-making process was employed when participants were not able to provide consent on their own, in which case both the participant and their substitute decision maker (SDM) were consulted, which occurred in 14/66 (21%) participants. The RA considered the cognitive capacity of participants (people with varying degrees of dementia/cognitive impairment) when explaining the study, its risks and benefits, and the consent process. The study was explained by the RA in a face-to-face discussion with the participant (and SDM when necessary) prior to the study session. The participant/SDM had 1 week to decide and respond to the request for consent. Ethics approval was obtained from the University Health Network Research Ethics Board (UHN REB), and separately from the ethics bodies that govern the various clinical partners.

#### Eligibility Criteria

Participants were *included* if they were (1) adults over 18 years old; (2) could communicate in English; and (3) were able to consent or had an SDM who could legally consent for them to participate in the study. Individuals were excluded if they met any of the following criteria: (1) vision impairment at a level that would make it impossible for them to see the VR films, as determined by the healthcare providers in the participant's circle of care at each site; (2) open wounds or skin conditions on the face, or chronic neck pain/injury that might make it unsafe to wear the VR HMD; (3) inability to provide consent and has a Public Guardian and Trustee as their SDM.

#### Demographics

We present the demographics by study site in Table 1. Across all sites, 66 participants were recruited, had a mean age of 80.5 years (based on 63 participants for whom we could obtain their age), and 60.6% of the sample were female. Thirty-nine percent of participants were married, 45% had a Bachelors degree or Post-graduate degree. Nine participants wore hearing aids during the VR experience. Fifty participants reported wearing glasses for any purpose (near and/or distance correction) and seventeen of these participants chose to wear their glasses during the VR experience. From a mobility perspective, nearly half of participants (31/66) were in a wheelchair during the VR experience, three were in their bed, and 32 used the chair designated for the study. Fifteen of the 66 participants had limited head mobility and 39 had limited body mobility Of the 66 participants, 60 were assigned a cognitive score based on their performance assessed with validated tools as per standard of care at each recruiting site: the Mini-Mental State Examination (MMSE) was used at Runnymede, the Montreal Cognitive Assessment (MoCA) at Baycrest, and the Cognitive Performance Scale (CPS) at Kensington. Dotsa Bitove Well-ness Academy does not formally assess cognitive status in their members and did not agree to have the research team assess them, therefore participants from this site did not have cognitive scores reported. Twenty-eight (47%) of the participants assessed had normal levels of cognition (scored >26 on MMSE or >25 on MoCA). Thirty-two (53%) of participants assessed presented with some degree of cognitive impairment [mild (*n* = 17), moderate (*n* = 12), or severe (*n* = 3)]. During their study session 20% of participants were accompanied by one caregiver (four were children, three were spouses, one friend, one private caregiver, and one sibling), and the remaining 80% of participants did not have caregivers with them.

**Table 1:** Distribution of study participants from each of the four study sites and their demographics.

	**Study Site**				**Total**
**Demographic variable** ** (*n*, %)**	**Baycrest *N* = 18 (27.27)**	**Kensington *N* = 33 (50.00)**	**Runnymede *N* = 10** ** (15.15)**	**Bitove *N* = 5** ** (7.58)**	**All *N* = 66** ** (100.00)**
**SEX (*****n*****, %)**					
Male	9 (50.00)	12 (36.36)	4 (40.00)	1 (20.00)	26 (39.39)
Female	9 (50.00)	21 (63.63)	6 (60.00)	4 (80.00)	40 (60.61)
**AGE (MEAN, SD)**					
	79.5 (9.1)	80.7 (11.7)	82.7 (10.1)	78.7 (8.8)	80.5 (10.5)
#With Age unknown	1	1		1	3
**MARITAL STATUS (*****n*****, %)**					
Married	11 (61.11)	8 (24.24)	6 (60.00)	2 (40.00)	26 (39.39)
Widowed	6 (33.33)	10 (30.30)	3 (30.00)	0 (0.00)	19 (28.79)
Divorced	0	4 (12.12)	0 (0.00)	1 (20.00)	4 (6.10)
Separated	1 (5.55)	2 (6.06)	0 (0.00)	1 (20.00)	4 (6.10)
Single	0	6 (18.18)	1 (10.00)	0 (0.00)	7 (10.60)
Unanswered/ Other	1 (5.55)	3 (9.09)	0 (0.00)	1 (20.00)	6 (9.10)
**Cognitive Impairment (CI) Level based on (MOCA/MMSE/CPS scores;** ***n*****, %)**	**CI mapped from MoCA score**	**CI mapped from CPS score**	**CI mapped from MMSE score**		
Normal	7 (38.89)	16 (48.48)	5 (50.00)	0 (0.00)	28 (42.42)
Mild	8 (44.44)	8 (24.24)	1 (10.00)	0 (0.00)	17 (25,75)
Moderate	2 (11.11)	9 (27.27)	1 (10.00)	0 (0.00)	12 (18.18)
Severe	0 (0.00)	0 (0.00)	3 (30.00)	0 (0.00)	3 (4.54)
Unknown	1 (5.56)	0 (0.00)	0 (0.00)	5 (100.00)	6 (9.09)
**AIDS (*****n*****, %)**					
Use glasses	18 (100.00)	24 (72.72)	5 (50.00)	3 (60.00)	50 (75.76)
Hearing difficulties	6 (33.33)	4 (12.12)	1 (10.00)	1 (20.00)	12 (18.18)
Wheelchair user	1 (5.55)	19 (57.57)	10 (100.00)	1 (20.00)	31 (46.96)
**MOBILITY (*****n*****, %)**					
Limited/no head mobility	1 (5.56)	11 (33.33)	3 (30.00)	0 (0.00)	15 (22.72)
Limited/no body mobility	5 (27.78)	25 (75.75)	8 (80.00)	1 (20.00)	39 (59.09)
**EDUCATION (*****n*****, %)**					
Elementary school	1 (5.56)	2 (6.06)	0 (0.00)	0 (0.00)	3 (4.54)
High school/equivalent	3 (16.67)	6 (18.18)	3 (30.00)	0 (0.00)	12 (18.18)
College	1 (5.56)	8 (24.24)	3 (30)	0 (0.00)	12 (18.18)
University/Bachelor's	7 (38.89)	9 (27.27)	2 (20.00)	1 (20.00)	19 (28.79)
Post-graduate degree	6 (33.33)	6 (18.18)	0 (0.00)	0 (0.00)	12 (18.18)
None	0	0	2 (20.00)	–	2 (3.03)
Unanswered	0	2 (6.06)	0 (00.00)	4 (40.00)	6 (9.09)

### Stimuli and Apparatus

#### Stimuli

**Figure 1:**
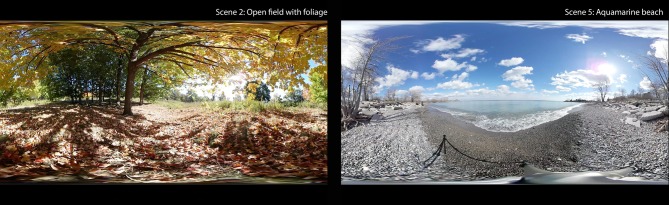
2D screen capture of two of the five VR scenes (Scenes 2: Open field with foliage and 5: Aquamarine beach).

A Samsung 360-degree camera was used to create custom VR films. The front and rear lenses of the camera each capture 180 degrees horizontally and vertically, creating a seamless and complete 360-degree field of view. Equipped with bright f2.0 Lens, visual scenes are recorded in 3,840 × 1,920 pixels (nearly 4K) high resolution. A collection of nature-based, live-action 360-footage was selected for the VR content (40). Five different scenes (45 s to 3 min each) were presented sequentially, resulting in a VR experience lasting 6 min in total. This 6-min video was automatically replayed from the beginning once all five scenes were experienced. Scene 1 featured a rocky shore, and waves; Scene 2 featured an open field with various colored foliage blowing in a gentle autumn wind; Scene 3 featured a dense forest with tall pine trees swaying in the wind; Scene 4 featured a black stone beach and ice water waves surrounded by a tall glacier, Scene 5 ut an aquamarine beach with gently flowing waves, bright blue sky and a family with a child and dog in the distance (see Figure 1 for a screen shot from Scenes 2 and 5).

#### VR Apparatus

**Figure 2:**
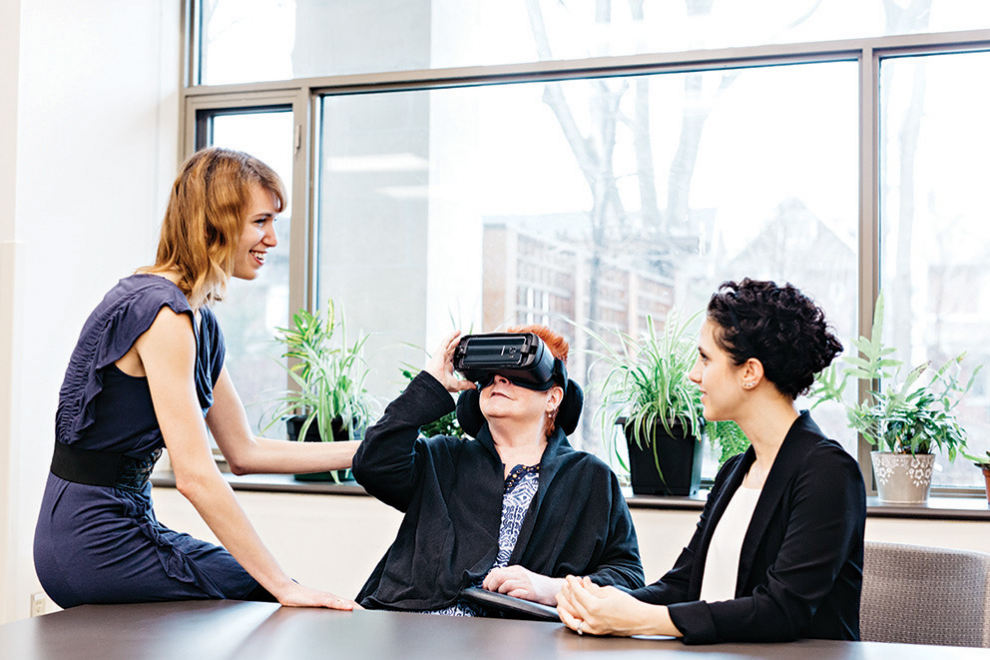
Participant tries the VR experience at one of the clinical sites with her caregiver. Written, informed consent was obtained from the individuals for the publication of this image. Runnymede Healthcare Center is credited for the photo and permission must be obtained for use in other sources.

The VR hardware system consisted of the following components: (1) Samsung S7 smartphone to view the VR films, (2) Samsung VR HMD that housed the smartphone/viewing screen and restricted the view of the real world, (3) Sennheiser HD 221 headphones to present the sound of the films and minimize the sound of the surrounding environment, and (4) VRology sanitary replaceable face-pads for individual use. The Samsung Gear VR HMD is a housing unit for the Samsung Galaxy phone, which is used to present the immersive VR experiences (i.e., the 360-degree VR films). The HMD weighs 318 grams, 60 Hz max refresh rate, offers a 101-degrees field of view, and is dark tinted to reduce glare and reflections. The headset itself has a pair of Oculus-made lenses, two buttons that allow for menu navigation, a navigational trackpad, and volume control. For every participant, the adjustment wheel at the top of the headset was used to calibrate the distance between the viewer's eyes and the lenses for improved focus. For hygiene purposes, each participant used their own personal VRology micro-fleece face-pad which avoided direct contact between the HMD and the user's face (41). The Samsung Gear VR was selected for this feasibility study due to its good technical specifications, being more hygienic (as the plastic casing could be disinfected easily when compared to fabric-based HMDs), and for accommodating eyeglasses. It was also more affordable compared to other systems of similar quality, was compatible with multiple types of smartphones and was able to access more freely available content (e.g., YouTube VR films). These characteristics make it potentially more scalable for broader adoption past the study completion. Figure 2 shows a participant trying the VR experience at one of the clinical sites with her caregiver.

### Study Procedure

#### Intervention Settings

Each of the four sites reserved a dedicated room to conduct individual VR sessions, which lasted 1 h on average. For three of the participants the intervention was administered in their private rooms due to mobility challenges or anxiety. The VR intervention was always administered while the participant was either seated or lying down, but never while standing up for safety reasons. Three participants received the VR intervention while lying in a reclined position in their bed, 31 participants received the intervention while seated in their own wheelchair and 32 participants received the intervention while seated on a dedicated swivel chair (with a floor-fixed base). Having the option to swivel/move allowed for a greater range of head/body motion when exploring the virtual environments. Once comfortable, the RA assisted the participant with putting on and adjusting the VR HMD and headphones and, once ready, launched the VR experience. The RA followed a detailed guide to observe the participant during their VR exposure and took notes of the participant's verbal remarks and expressions. The participant could watch the entire VR-experience repeatedly until a maximum of 20 min, at which point the RA concluded the exposure session. The participant could also stop at any time by indicating their desire to stop verbally or by removing the HMD. VR study sessions were scheduled at a preferred day/time that could accommodate the participant's and staffs' preferences and that avoided conflicts with site programs.

#### Measurement Tools

##### Demographics

Some demographic information was obtained through chart review (age/sex), while other demographic and health history information was provided by participants at the start of the study session including level of education, marital status, proneness to motion sickness, nausea or dizziness, hearing and vision impairments, mobility limitations or use of physical aids, and previous experiences using VR. Cognitive status and scores on standardized tests of cognition (MMSE, MoCA, CPS) were obtained from chart reviews. Since the different sites did not use the same cognitive assessment tool, a mapping scheme was used to standardize the different scores into four categories: normal, mild, moderate, and severe cognitive impairment (see Table 2). With respect to the experimental measures of interest, we used several quantitative and qualitative measures to collect information from participants before, during, and after their VR experiences (described below).

**Table 2:** Mapping MoCA, MMSE, CPS scores to normalize cognitive impairment levels.

**CI level**	**MoCA scores**	**MMSE scores**	**CPS scores**
Normal	26–30	27–30	0–1
Mild	11–25	18–26	2
Moderate	6–10	10–17	3–4
Severe	<6	<10	5–6

##### Pre-intervention measures

A modified version of the State-Trait Anxiety Inventory (STAI) (42), a validated questionnaire using Likert-scales (1-5), was used to collect information about participant's current state of anxiety. Both pre- and post-intervention questionnaires were administered verbally and participants were asked to respond when they were able to.

##### During intervention measures

As an overall measure of VR experience tolerability, we reported on the percentage of participants who were able to view the entire film once and recorded how long participants kept the HMD on and viewed the films. A modified version of the Music in Dementia Assessment Scales (MiDAS) questionnaire, developed and validated to evaluate music-therapy for people with dementia (43), was completed by the RA to assess whether there were observable changes in the participant's mood/behavior and engagement (e.g., interest, response, initiation, enjoyment) while exposed to VR-therapy. Two RAs followed the scripted instructions and independently recorded (through written notes that were later transcribed) their observations of the participant during VR exposure. The script included observations about any vocal utterances, facial expressions, and body movements made by the participant. Researchers also completed a scale evaluating the participant's level of enjoyment interpreted through observations of reactions and/or elicitation of spontaneous conversations such as recounting stories or pleasant life memories. Additionally, RAs recorded participant's comments and observations regarding the VR device comfort.

##### Post-intervention measures

Participants completed the same modified STAI questionnaire as was administered pre-intervention as a way of measuring any changes in anxiety following VR exposure. Participants also completed a custom-developed Likert-scale questionnaire consisting of several open-ended questions regarding the VR experience. Specifically, they were asked to rate on a scale from one (strongly disagree) to five (strongly agree) a number of statements concerning their, comfort, emotional state and subjective well-being while experiencing VR. Participants were asked about any discomfort or inconveniences experienced during the process of fitting the VR HMD, whether the HMD was too heavy, if it applied too much pressure on their head or face. Open ended questions were used to capture any other discomfort experienced by participants. Several questions in the post-intervention survey addressed characteristics of the VR films: the fidelity of the image, sound quality, length of the films, overall exposure, and diversity of scenes.

To measure changes in feelings of enjoyment triggered by VR exposure, we recorded the number of decreased negative emotional states, and the number of increased positive emotional states post- vs. pre-intervention. A number of questions posed pre- and post-VR exposure asked about the emotional state of participants (using a 5-point Likert scale). See Supplemental Material for the data collection tool.

## Results

### Comfort, Tolerability, and Side-Effects of VR

Every individual who consented followed through and participated in the study with a 0% dropout rate. The average time that participants spent viewing the immersive VR experience was 8 min, with a minimum of 3 min, and a maximum of 20 min exposure before the HMD was removed. Fifty participants (76%) completed at least one full round (the entire 6 min) of VR experience. In terms of participants' tolerance to the hardware, 88% (58/66) responded “no” to the question “Did the VR HMD feel too heavy?” and 82% (54/66) found the VR HMD easy to get used to; one participant commented that he “*forgot [he] had it on”* another said, “*I didn't even notice when I had it on.”* Some (8%) found the device somewhat or too heavy, although one participant who rated it as heavy added that this was “*worth the mild discomfort.”* An RA observation of a participant indicated that despite a “runny nose,” the VR HMD didn't bother her; at first she wanted to hold the HMD with both hands because whenever she feels pressure on her nose, she gets claustrophobic/panicky, but as the film progressed it became more comfortable. Eighty-five percent of participants (56/66) responded “yes” to being able to move their head up and down and side-to-side easily, and of the participants who were in a chair or wheelchair (*N* = 63), 65% felt comfortable moving around with the swivel-chair/wheelchair to see more of the surroundings in the films. In terms of the image/display quality, 25% of participants found that it was challenging to view because the image was not “in focus.” The HMD had some capability for focus adjustment, but appeared to be insufficient, especially for individuals wearing glasses.

In order to determine potential negative side effects related to physical well-being and interference with medical devices, we collected baseline information regarding participants' susceptibility to motion sickness, nausea and dizziness, and recorded whether they were using hearing aids while in VR. Ninety-two percent of participants (58/63) reported not feeling nauseous during the VR experience. Of the 22% of participants who reported being prone to dizziness and nausea at baseline, none experienced these symptoms during or after the VR exposure. Of all the participants who wore hearing aids during the VR session, none reported any issues with their devices (such as buzzing or static noises) due to interference with the VR HMD.

When asked about the length of the different films included in the VR experience and overall exposure time, 80% (53/66) of participants reported that the length of films was appropriate and “adequate for immersion,” but some (6%) wanted the exposure time to be even longer. Table 3 summarizes the number of responses and average ratings on a 5-point Likert scale, from 1 (strongly disagree) to 5 (strongly agree) on questions relating to the interest, immersiveness, and quality of entertainment experienced while in VR.

**Table 3:** Ratings of enjoyment, engagement, and immersion following VR exposure.

**Questionnaire Statement**	**Average rating**
There is nothing worth looking at in these videos	1.42 (59)
Watching this was boring	1.47 (62)
You became so involved that you were no longer aware of my real environment (Of things happening around you)	2.41 (58)
This helped you relax and get relief from unwanted feelings or thoughts	3.34 (62)
You had a lot of fun watching this	3.49 (62)
You want to spend more time looking at these surroundings	3.55 (60)
There was much to explore and discover	3.57 (58)
Watching this was fascinating	3.59 (59)
Your attention was drawn to many interesting things	3.91 (58)
You would like to see more places like these	4.19 (58)
The virtual world seemed very real to you.	4.21 (62)

*Ratings for each item ranged from 1 (strongly disagree) to 5 (strongly agree). The total number of responses are included in brackets*.

For the post-intervention survey, the most common response showed strong disagreement for the following questions (1) Did you feel there was too much going on? (51/66 strongly disagreed and 2/66 disagreed) (2) Did you feel panicked while watching? (56/66 strongly disagreed and 3/66 disagreed) (3) Did you feel like you wanted to get out of the situation? (50/66 strongly disagreed and 4/66 disagreed) and (4) Did you feel confused or disoriented? (54/66 strongly disagreed and 1/66 disagreed). Throughout all intervention sessions and across all participants, no additional or unexpected adverse events or reactions were reported by the participants or witnessed by the RAs. One participant commented that *it “could… [get] too intense if [one] were to wear this for a long time and get fully immersed… [like] a 3D/4D science center movie that was unbearable after too long.”*

### Enjoyment of VR

**Figure 3:**
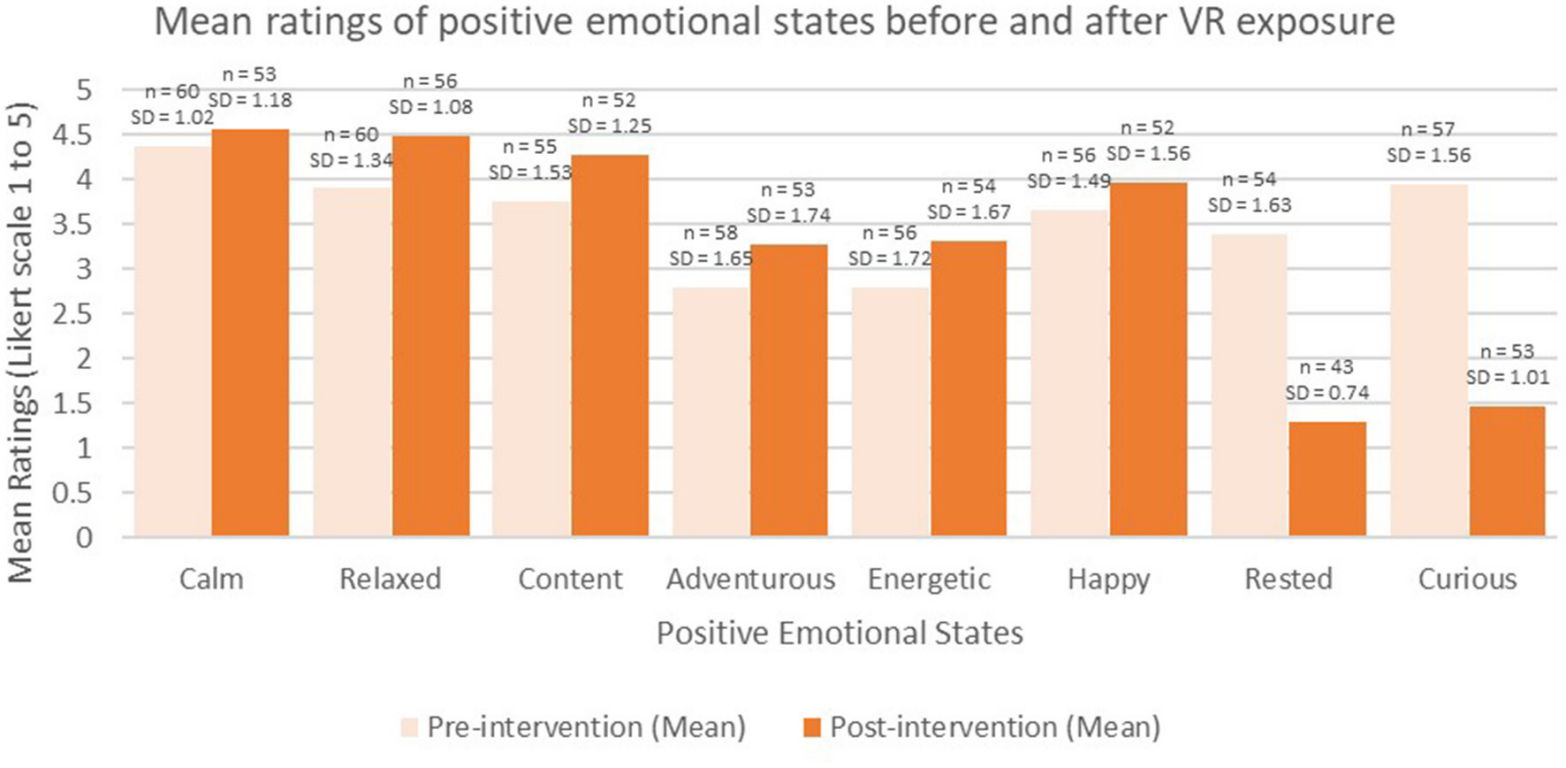
Mean ratings of positive emotional states before and after VR exposure.

**Figure 4:**
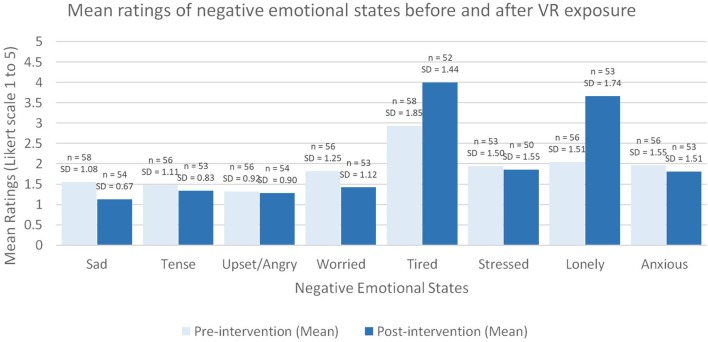
Mean ratings of negative emotional states before and after VR exposure.

See Table 4 for the participants responses to the emotional states pre- and post-intervention. Of the sixteen “emotional modality” items that were measured pre- and post- VR exposure, half of the items were associated with positive effects on emotion if the values on the rating scale increased (e.g., happy; shown with white background in Table 4), and half of the items were associated with negative effects on emotion if values on the rating scale increased (e.g., sad; shown in a shaded background in Table 4). In terms of the positive emotions, all but two items increased (“rested” and “curious” decreased) (see Figure 3) and none of the negative items apart from two items increased (“lonely” and “tired” increased) (see Figure 4).

**Table 4:** Pre/Post VR emotional state questions (based on a modified STAI tool).

**#**	**Emotional modality**	**Pre-intervention** ** (Mean, # responses, SD)**	**Post-intervention** ** (Mean, # responses, SD)**	**Change in emotional state**
1	Calm	4.37 (60, 1.02)	4.57 (53, 1.18)	Increase
2	Relaxed	3.9 (60, 1.34)	4.48 (56, 1.08)	Increase
3	Content	3.76 (55, 1.53)	4.27 (52, 1.25)	Increase
4	Adventurous	2.79 (58, 1.65)	3.28 (53, 1.74)	Increase
5	Energetic	2.79 (56, 1.72)	3.31 (54, 1.67)	Increase
6	Happy	3.66 (56, 1.49)	3.96 (52, 1.56)	Increase
7	Rested	3.39 (54, 1.63)	1.30 (43, 0.74)	Decrease
8	Curious	3.95 (57, 1.56)	1.47 (53, 1.01)	Decrease
9	Sad	1.55 (58, 1.08)	1.13 (54, 0.67)	Decrease
10	Tense	1.48 (56, 1.11)	1.34 (53, 0.83)	Decrease
11	Upset/Angry	1.32 (56, 0.92)	1.28 (54, 0.90)	Decrease
12	Worried	1.82 (56, 1.25)	1.42 (53, 1.12)	Decrease
13	Tired	2.93 (58, 1.85)	4.00 (52, 1.44)	Increase
14	Stressed	1.94 (53, 1.50)	1.86 (50, 1.55)	Decrease
15	Lonely	2.04 (56, 1.51)	3.66 (53, 1.74)	increase
16	Anxious	1.96 (56, 1.55)	1.81 (53, 1.51)	Decrease

*Ratings for each item ranged from 1(a little) to 5 (a lot)*.

From the standardized RA observations, it was found that 50% of participants tried to interact with the virtual environment by moving and looking around during the exposure despite physical impairments (e.g., limited mobility), and 40% were very expressive including positive expressions of enjoyment, commentary, and conversation. Most participants (79%) reported that the virtual world seemed very real, and 73% wanted to view “more places like these.”

Some comments by participants included the following:

“*It was like you were there, sitting by the water; I kept wanting to stretch my feet out, get them wet – ha! I would have stayed [there] forever. It wasn't long enough*.”

“*A person could be there for* ½ *hour or an hour that would be wonderful for the people that cannot get anywhere on their own, I think. Marvelous*.”

“*I would see a lake… and we would go there as kids and there were fields and we'd have walkways through it and I picture myself being there to relax … I relate to it*.”

When asked about their enjoyment of the content of the VR films, several found the experience slow-paced or missing certain elements such as animals or pets, people, city-life, and adventure, and wished there were more engaging themes, even if they would not be so relaxing. For example, one participant expressed, “*There were no mountains - I would like mountains, and valleys and stuff like that. I could do with a good big storm, yeah, a crashing wave. [It] might not be relaxing, but it would be funny for me – it has to be interesting too! These are nice ideas.”*

Participants commented that watching the same films of landscape multiple times had the potential to become redundant. A consistent theme in participants' responses was the need for more variability in the choice of films available in order to accommodate different participant's personal preferences. For example, some participants expressed their wish to see city-life instead of forests, and some described how meaningful it would be to see their hometown or other familiar, real-world places as a way to remember the past.

Table 5 depicts the presence and intensity of participants' reactions or interactions during the VR experience as observed and rated by the RA. Seventy-six percent (50/66) exhibited some or substantial alertness deduced from the changing of their posture (e.g., showing desire to see more by moving around/touching), and 55% (36/66) had some or substantial facial expression changes. Sixty-five percent (43/66) initiated conversation or made vocalizations during the VR experience, and the RA rated 61% (40/66) of participants as exhibiting either some or substantial levels of enjoyment during VR exposure, deduced from participant's reactions such as smiling, laughing, brighter mood, playfulness, sense of humor, and relaxed mood.

**Table 5:** The number of participants who observed having no, little, some, or substantial reactions while experiencing VR (based on a modified MiDAS tool).

**Question**	**None**	**Little**	**Some**	**Substantial**
Did the participant's posture indicate his/her awareness while in VR? (e.g., showing desire to see more by moving around/touching)	1	14	19	31
Did the participant's facial expression indicate his/her awareness while in VR?	11	17	20	16
Did he/she initiate conversation or make vocalization that showed interest? (For example, ooh's, ah's, giggling, or saying “wow”)	13	7	21	22
Did he/she talk about his/her life experiences (reminiscence) or mention memories meaningful to them?	37	6	11	5
Rate the participant's level of enjoyment during communication/activity. For example: Smiling, laughing, brighter mood, playfulness, sense of humor, relaxed mood	13	7	22	18

In addition to the enjoyment observed, about a third of all participants (23/66) engaged in conversation related to what they were experiencing while in VR, a third (22/66) recalled memories from their past, and some wanted to share the experience with others by handing the VR HMD to their caregiver and asking them to also watch the VR experience. Half of all participants (51%) indicated that the VR experience helped them gain relief from unwanted feelings or thoughts. Importantly, most participants (76%) expressed a desire to try VR again and 71% indicated that they would recommend the VR experience to a friend.

## Discussion

The primary purpose of the current study was to determine if it is feasible and of benefit to provide VR experiences to variably dependent older adults living in long-term care or receiving out-patient care due to impairments in sensory, motor, and/or cognitive functioning. Although new technologies are often received with skepticism and resistance in healthcare, this feasibility study was easily accepted at all four sites. Feasibility and acceptability were also reflected by the fact that multiple sites (with different staff, unique resources, and varying environments/cultures) were keen and willing to embark upon this research project. Even though each of the four sites have their own recreational therapy programs and dedicated outdoor environments accessible to residents, they were very interested in exploring novel ideas to engage their clients in broader experiences. Overall, results were positive, as the intervention was well-tolerated by individuals with various sensory, cognitive, and physical health conditions including individuals who use glasses, hearing aids, and mobility devices such as wheelchairs, as well as individuals who had clinically significant cognitive impairments. The fact that the results did not appear to be unique to particular sites, or site characteristics (e.g., LTC vs. day-center) speaks to the potential generalizability of the findings.

VR exposure also did not cause adverse side effects (nausea, dizziness, disorientation, confusion) and was generally considered to be quite enjoyable. Overall, compared to pre-intervention, participants reported feeling more energetic, content, relaxed, and adventurous, and less anxious, worried, or stressed after the intervention. Most participants expressed a desire to try VR again and would recommend the VR experience to a friend.

It is interesting to note the change in confidence regarding VR by the site study coordinators. All participants exhibited some degree of age-related declines in sensory and motor function, and just over half of the participants were diagnosed with cognitive impairment, whereas 42% were cognitively normal. This was likely due to increased caution exhibited by study coordinators at the clinical sites, who were the ones to identify and approach potential participants. Over the course of the feasibility study, preliminary evidence about lack of side effects and the positive outcomes contributed to healthcare professionals becoming increasingly more knowledgeable and confident regarding using the VR technology with increasingly impaired patients.

### Comfort, Tolerability, and Side-Effects of VR

While VR has been studied in other clinical populations, researchers and caregivers have been cautious not to generalize its presumed benefits to the impaired older adult population, given that this population may have unique preferences, as well as specific safety and tolerability needs. Our results, however, found that most participants tolerated the hardware and software content very well and did not report any adverse side effects due to either simulator sickness (e.g., nausea, disorientation) or due to confusion or anxiety. In fact, ninety-six percent of participants, including those who self-identified as being prone to dizziness and nausea at baseline (22%), did not experience any adverse events. Most participants (85%) described the HMD as easy to accommodate to, and even those who found the device heavy still found the comfort acceptable.

Although initially we had concerns that limits to mobility and range-of-motion in this population may prevent them from experiencing the full benefits that can be realized through exploring the 360-degree scene with head and body movements, we found, that despite various mobility problems, most participants (90%) were able to move their heads side-to-side and up and down in order to observe much of the VR surroundings. Seventy-five percent of participants who used the swivel chair felt comfortable rotating the chair and participants who were sitting in their own wheelchairs could request that a caregiver or an RA turn their wheelchair.

Wearing corrective eyeglasses was problematic for many of the participants, both in terms of fitting the HMD over the glasses and with achieving focus and clarity when viewing the VR films. Visual declines become more common and more pronounced with older age and 30–57% of older adults living in LTC have a visual impairment (44). Therefore, to be successful with this population the hardware must provide adequate support for users who wear glasses. Another unanticipated barrier to using the HMD was related to the discomfort expressed by participants who were wearing wigs and aesthetic concerns about disturbing one's hairstyle.

Among the health and safety warnings listed by the HMD manufacturer is that hearing aids may be affected by Radio Frequency interference from the HMD. Given that more than 80% of adults 85 years of age and older have hearing loss (45), and many of them are hearing aid users, this could be a serious concern. We found, however, that none of the participants who wore hearing aids during the VR session reported any issues with their devices (such as buzzing or static noises). That said, the simulated auditory content in the current VR scenarios included very simple, ambient soundscapes and it remains unknown whether VR scenarios that involve more complex sounds or speech-based content would be more adversely affected by this potential interference. This will be an important consideration when developing novel VR hardware and content for any population using hearing aids.

#### Characteristics of the VR Content

Given that there are no precedents in the literature regarding the types of VR scenes and experiences that are preferred by older adults within these settings, we took a conservative approach in the current study to limit the experiences to mildly stimulating, calming scenes from nature. This was done to exploit the known benefits of exposure to nature shown in previous literature, but also to avoid introducing older adults with experiences that might be too overwhelming, intimidating, or confusing. This was a particular concern in individuals with moderate to severe cognitive impairments. The results demonstrated that when participants were asked about the entertainment value and engagement in the experiences their highest average ratings (i.e., strongly agree) were assigned to positive items (e.g., “the virtual world seemed very real” and “I would like to see more places like these”) and the lowest average ratings (i.e., strongly disagree) were assigned to the negative items (e.g., “watching this was boring” and “there is nothing worth looking at in these videos”). That said, participants did express interest in experiencing different types of VR experiences than those included in the study, with recurring themes including a preference for more dynamic scenes, more social scenes (people and animals), and familiar real-world scenes (i.e., places from their history). The majority of participants (84%) felt that the duration of the VR films (four of them being 45 s in length, and the fifth being 3 min in length; together as a complete set 6 min in total) was adequate and half of them expressed losing track of time while experiencing the VR films. Two thirds (44/66) of all participants engaged in the VR experiences longer (between 7 and 20 min) than the prescribed time (6 min).

From these findings we can ascertain that older adults can be immersed safely, and with interest for an average of 8 min (and up to 20 min). Perhaps the reason that the average viewing time was 8 min, just over the 6 min total length of the combined films before they looped back and repeated, is that participants saw things they had already seen before, and this made them take the HMD off sooner than if new material was presented. Future studies should experiment with new vs. repeated content, for the same overall length of VR experiences, to determine if the viewing time can be increased if novel (unrepeated) content is presented. Moreover, studies should look at how the VR viewing time is affected when customized content is presented as desired by the individual participants. In their systematic review, Benjamin et al. reported that a common barrier to outdoor engagement in LTC settings is the “one-size-fits-all” approach to activity design, which leaves residents of different functional abilities dissatisfied with programming that does not match their capabilities (21). In contrast, VR creates an opportunity to customize experiences for individuals in an efficient and cost-effective way. Future studies should incorporate a wider variety of VR experiences, to provide an opportunity to accommodate individual preferences, while also considering cognitive, sensory, and mobility constraints.

Another important observation that was frequently recorded during the VR experiences was that the participants become engaged with the other people involved in the session, including their family members, caregivers, and study staff. They were keen to describe their experiences in the moment, as well as to reminisce about other memories that were provoked by the VR experience. Some participants also wanted to directly share the VR experience with family members who were present, by asking them to put on the HMD to view the scenes. This was a very important observation that emphasizes the potential for VR to help increase social engagement through experiences that evoke sharing and storytelling. It also indicates that future VR interventions should consider providing joint or multi-user experiences (e.g., multi-display set ups).

### Enjoyment of VR

Results demonstrated that, on average, participants experienced increases in levels of positive emotions (such as feeling relaxed, and content) and decreases in levels of negative emotions (such as feeling sad or anxious) after VR exposure. Among the unexpected results was participants' reports of feeling less “rested” and more “tired” after the VR session. While it is possible that fatigue resulted from being in the actual VR HMD, we suspect it was also associated with the relatively lengthy study protocol prior to trying on the HMD (e.g., questionnaires and set-up which took ~30 min). Interestingly, feeling adventurous reportedly increased, while feelings of curiosity decreased after VR. The decrease in feeling “curious” may have been in response to post intervention fulfillment of initial interest in the new technology (i.e., before exposure being “more curious” and after being “less curious”).

Of interest was the average increase in levels of feeling lonely post intervention, given that a desirable outcome of VR is reduction in feelings of isolation. Participants did not elaborate as to why they felt “more lonely,” however some participants expressed their desire to give their caregivers a chance to see what they had just experienced. The VR experience effect on feelings of loneliness in this population should be studied further.

In over 50% of participants, the research team observed increases in awareness, changes in posture, facial expression, and movement while in VR, compared to during pre-VR discussions. Perhaps the strongest evidence of enjoyment was revealed in the qualitative, open feedback participants provided, and countless unsolicited exclamations such as “*this is wonderful”, “loved it”, “this is so beautiful.”*

## Limitations

As this was a feasibility study meant to primarily evaluate the side effects and tolerability of VR, it was non-randomized and employed a purposeful sampling technique. Research site coordinators approached potential participants who they felt might be open to this type of new technology, and could benefit from VR exposure; thus, there was bias toward acceptance, which is characteristic of individuals willing to try new devices and technologies. Although this may limit generalizability, candidates for VR exposure therapy would also be individuals open to new treatment approaches and willing to try new therapies and devices; hence the study sample population may in fact be more representative of the actual prospective user population.

In the current study, we measured outcomes based on participant ratings, and unblinded researchers' observations, in order to evaluate impact on symptoms and emotional states. Also, the study did not have a control group or control condition against which to compare the VR effects. Adding biophysiological measures such as cortisol stress responses, heartrate, and skin conductance, as well as implementing a randomized controlled trial design will increase the validity and interpretation of the outcomes.

## Future Directions

This study provided novel insights and identified priority areas for further study of the potential benefits of VR exposure for variably dependent older adults. Future studies should expand research into different types of healthcare institutions and community care, from acute-care hospitals to private homes to see where VR can be best and most easily provided. Consideration for sub-group analyses is also warranted.

Our findings support the need to further investigate VR as a tool to enhance social-emotional behavior among people with physical impairment and dementia. VR may have distinct effects on individuals with sensory and/or motor and/or cognitive abilities. For example, VR-therapy may be evaluated as a means to managing behavioral and psychological symptoms of dementia during acute hospitalization.

Particular interest should be given to people with more advanced stages of dementia (moderate to severe), as there are challenges in managing symptoms and improving quality of life in these individuals using non-pharmacological interventions. It would be very interesting to look at this group separately to see if their reactions to VR-therapy are consistent with others. Of all participants tested, arguably it is this group that has the most unknowns regarding tolerance and engagement.

In addition to gaps in knowledge regarding customizations of VR therapies based on setting and population, questions are raised in terms of ideal exposure length and frequency. While we documented immediate effects after one short exposure session, it is uncertain as to the duration of these effects and whether there is a “dose effect” (i.e., more is better up until some cut-point). Finally, there is great interest in developing and evaluating if more dynamic, social, reminiscent, interactive, multisensory, and personalized VR content would have even greater positive effects.

## Conclusion

The results of our study show that being exposed to immersive VR using an HMD is a feasible, safe approach to providing beneficial experiences to older adults with mobility, sensory, and/or cognitive impairments. Participants tolerated the VR hardware, were able to physically explore the virtual environments through head/body movements and did not report any adverse side effects. For the participants in this study, who were already more open to new interventions, the VR experience seemed to have a positive impact on their mood; the majority reported positive emotional changes following the VR session, were enthusiastic about trying VR again and would recommend the experience to others. The success of having implemented the study across diverse health care settings also speaks to the potential for broad implementation.

## Data Availability Statement

The datasets generated for this study are available on request to the corresponding author.

## Ethics Statement

The studies involving human participants were reviewed and approved by University Health Network Research Ethics Board. The patients/participants provided their written informed consent to participate in this study.

## Author Contributions

LA and EA were responsible for the conception and design of the study. LA was responsible for conducting study site visits, collecting data, and writing the first draft. EA was responsible for analyzing the data and editing the manuscript. OB helped draft the first version of the manuscript. EA, LC, MW, NE, and OB were instrumental to study site visits and conducting observations. HA helped revise the protocol and edit the manuscript. JC was responsible for revising the manuscript critically for important intellectual content.

### Conflict of Interest

The authors declare that the research was conducted in the absence of any commercial or financial relationships that could be construed as a potential conflict of interest.
