# Supplementary material for: Older Adults With Cognitive and/or Physical Impairments Can Benefit From Immersive Virtual Reality Experiences: A Feasibility Study
Source: Front Med (Lausanne). 2020 Jan 15;6:329. doi: 10.3389/fmed.2019.00329 (PMC6974513; doi:10.3389/fmed.2019.00329)
Supplement: Supplementary file 1 [file Data_Sheet_1.PDF]

Prescribing VRx - Dementia (Version July 4, 2017)

Demographics

1. Name of Researcher(s)

2. Research Site

- Baycrest Health Sciences
- Kensington Health Gardens
- Runnymede Rehab
- Bitove Academy
- Other (please specify)

3. Participant #

4. MMS/MOCA Score

5. Record Date and Observation start time

Date / Time

MM/DD/YYYY hh mm -

Demographics

6. Sex

- ◯ Male
- ◯ Female

7. Age (Retrieve from chart)

8. What is/was your profession? (Note for researcher: First verbal question)

9. What fits most closely to your current living state?

- ☐ Married
- ☐ Divorced
- ☐ Separated
- ☐ Single
- ☐ Widowed
- ☐ Living with a partner/Spouse
- ☐ Living with family members
- ☐ Other (please specify)

10. What is your primary language? (The one you're most comfortable speaking)

11. What is the highest level of education you have completed

- ○ Elementary School
- ○ High School or Equivalent
- ○ College
- ○ University - Bachelor's Degree
- ○ Post-graduate Degree
- ○ Other (please specify)

Pre-VR Experience

Relaxing Experiences and Fun Activities

12. What do you do to relax? (How often do you get a chance to...)

Would you like some examples - Do you like to be in nature? Do you like to sit and watch people in a park or coffee shop, or shopping mall? Do you prefer calm, quiet, still places or active, lively, noisy places?

13. What do you do for fun? (How often do you get a chance to...)

Would you like some examples - Listen to music, walk, spend time with family, watch movies, watch sports, dance, concerts, board games, computer games?

14. When watching movies or shows, do you find that you easily become deeply involved?

Probe: Do you sometimes react to the scene or actors as if you were there? Or are you unemotional/unmoved when watching movies?

Pre-VR Experience

Previous Knowledge about VR

15. Did you hear about virtual reality technology before?

- ◯ Yes
- ◯ No
- ◯ Maybe
- ◯ Other (please specify)

16. Did you try a VR headset before? (VR head-mounted device)

- ◯ Yes
- ◯ No
- ◯ Maybe
- ◯ Other (please specify)

Pre-VR Experience

Nausea/Dizziness/Impairments

17. Do you easily experience motion-sickness/nausea?
(e.g. reading in a car, being in a boat, etc.)

- ◯ Yes
- ◯ No
- ◯ Somewhat

18. Do you feel dizzy right now?

- ◯ Yes
- ◯ No
- ◯ Somewhat

Pre-VR Experience

19. On a scale from 1-5 (1= not at all; 5= very much), do you feel:

	1	2	3	4	5
Calm	○	○	○	○	○
Sad	○	○	○	○	○
Tense	○	○	○	○	○
Upset/angry	○	○	○	○	○
Relaxed	○	○	○	○	○
Content	○	○	○	○	○
Worried	○	○	○	○	○
Adventurous	○	○	○	○	○
Energetic	○	○	○	○	○
Happy	○	○	○	○	○
Tired	○	○	○	○	○
Stressed	○	○	○	○	○
Rested	○	○	○	○	○
Lonely	○	○	○	○	○
Curious	○	○	○	○	○
Anxious	○	○	○	○	○

Observation

Participant Description

20. Head mobility

- ◯ Normal
- ◯ Difficult
- ◯ Almost immobile

Additional comments

21. Range of body motion

- ◯ Normal
- ◯ Limited
- ◯ Static

Additional comments

22. Do you wear glasses?

- ◯ Yes
- ◯ No

23. Do you have trouble hearing?

- ◯ Yes
- ◯ No

Please describe:

24. Assistive devices/accessories being worn

	Yes	No
Wearing glasses now	◯	◯
Hearing aids now	◯	◯

25. Other physical aids

26. Participant in wheelchair?

- Yes
- No

27. Family members/Caregivers present?

- Yes
- No

Other (please specify)

Observation

Family/Caregiver(s)

28. How many present?

29. Relationship to patient?

Observation

Levels of Interest in objects/activities/people around him/her

30. Before the VR experience, how much interest did the participant show in activities or other people's sounds around him/her?

- ◯ Substantial
- ◯ Some
- ◯ Little
- ◯ None

31. Please indicate the time that the participant STARTED watching the VR film.

Date / Time

[hh] [mm] [-]

32. Did the participant's posture indicate his/her awareness while in VR? (E.g. showing desire to see more by moving around/touching)

- ◯ Substantial
- ◯ Some
- ◯ Little
- ◯ None

33. Did the participant's facial expression indicate his/her awareness while in VR?

- ◯ Substantial
- ◯ Some
- ◯ Little
- ◯ None

Observation

Levels of Initiation and engagement in communication/activity

34. Did he/she initiate conversation or make vocalization that shows interest?
(For example, ooh's, ah's, giggling, or saying 'wow')

- ○ Substantial
- ○ Some
- ○ Little
- ○ None

Additional comments

35. Did he/she talk about his/her life experiences (reminiscence) or mention memories meaningful to them?

- ○ Substantial
- ○ Some
- ○ Little
- ○ None

Additional Comments

36. Rate the participant's level of enjoyment during communication/activity.
For example:

- Smiling, laughing, brighter mood
- Playfulness, sense of humor
- Relaxed mood

- ○ Substantial
- ○ Some
- ○ Little
- ○ None

Observation

Levels of Enjoyment during communication/activity

37. Please note any major reactions from the participant you may have noticed.

Use this list as supplementary information:

agitation/aggression Relaxed mood Withdrawn/low in mood

attentive/interested Restless/anxious Cheerful/Smiling

38. Please indicate the time that the participant FINISHED watching the VR film.

Date / Time

hh mm -

Post - VR Experience

39. On a scale of 1-5 (1= not at all; 5= very much), do you feel...

	1	2	3	4	5
Calm	○	○	○	○	○
Sad	○	○	○	○	○
Tense	○	○	○	○	○
Upset/angry	○	○	○	○	○
Relaxed	○	○	○	○	○
Content	○	○	○	○	○
Worried	○	○	○	○	○
Adventurous	○	○	○	○	○
Energetic	○	○	○	○	○
Curious	○	○	○	○	○
Happy	○	○	○	○	○
Tired	○	○	○	○	○
Stressed	○	○	○	○	○
Rested	○	○	○	○	○
Lonely	○	○	○	○	○
Anxious	○	○	○	○	○

Post-VR Experience

40. Indicate, on a scale of 1-5 (1=disagree; 5=agree), if you agree or disagree with the following statements:

	1	2	3	4	5
The virtual world seemed very real to you.	○	○	○	○	○
This helped you relax and get relief from unwanted feelings or thoughts	○	○	○	○	○
Your attention was drawn to many interesting things	○	○	○	○	○
You would like to see more places like these	○	○	○	○	○
There was much to explore and discover	○	○	○	○	○
You want to spend more time looking at these surroundings	○	○	○	○	○
Watching this was boring	○	○	○	○	○
You had a lot of fun watching this	○	○	○	○	○
Watching this was fascinating	○	○	○	○	○
There is nothing worth looking at in these videos	○	○	○	○	○
There was too much going on	○	○	○	○	○
You felt you panicked while watching this	○	○	○	○	○
You felt you wanted to get out of this situation or avoid it	○	○	○	○	○
You felt confused or disoriented	○	○	○	○	○
You became so involved that you were no longer aware of my real environment (Of things happening around you)	○	○	○	○	○

Post-VR Experience

41. Indicate whether you agree or disagree with the following

	Yes	Somewhat/Maybe	No	Not Sure
Were the films too long?				
Specifically, was the last film too long? (Beach)				
When watching did you feel like you lost track of time? (In the comments, indicate how long the participant THOUGHT they were watching for.)				
Did you find the VR headset easy to get used to?				
Did you find the VR headset too heavy?				
Did you feel nauseated while watching the VR?				
Could you move your head up and down, and side-to-side easily to see more of the surroundings in the films?				
Did you feel comfortable moving around with the chair to see more of the surroundings in the films				

Were the films too long?

Additional comments

Specifically, was the last film too long? (Beach)

Additional comments

When watching did you feel like you lost track of time? (In the comments, indicate how long the participant THOUGHT they were watching for.)

Additional comments

Did you find the VR headset easy to get used to?

Additional comments

Did you find the VR headset too heavy?

Additional comments

Did you feel nauseated while watching the VR?

Additional comments

Could you move your head up and down, and side-to-side easily to see more of the surroundings in the films?

Additional comments

Did you feel comfortable moving around with the chair to see more of the surroundings in the films

Additional comments

Post - VR Experience

42. Overall, what did you like most about the virtual reality experience?

43. Which was your favorite scene? How so?

44. What other places would you like to see in Virtual Reality?

45. Which was your least favorite film? How so?

46. Was there something that you did not like about the VR experience?

47. Would you like to own a VR headset at home?

48. Do you own a smartphone or iPad?

Post-VR Experience and Observation

49. Would you recommend this Virtual Reality experience to a friend?

- ○ Yes
- ○ No
- ○ Unsure

50. Would you want to try this experience again?

- ○ Yes
- ○ No
- ○ Unsure

Additional Comments

[ ]

51. Record observation end time

Date / Time

hh mm -

52. Additional comments

[ ]
